# A Rare Case of Delayed‐Onset Hypersensitivity Reaction and Complete Secondary Treatment Failure Following Repeated Cosmetic Botulinum Toxin Type A Injections

**DOI:** 10.1111/jocd.70145

**Published:** 2025-04-02

**Authors:** Yan Bian, Chi Zhang, Shaohua Wang, Lili Zhang, Hong Cai

**Affiliations:** ^1^ Department of Dermatology, Air Force Medical Center PLA Beijing People's Republic of China; ^2^ Beijing Integrated Traditional Chinese and Western Medicine Hospital Beijing People's Republic of China

**Keywords:** adverse effects, botulinum toxins, type A, cosmetic procedures, skin hypersensitivity reaction, treatment failure

## Abstract

**Background:**

Cosmetic botulinum toxin type A (BTX‐A) injections have been widely used for improving facial aesthetics. Although the procedure is generally safe, immune‐mediated adverse events, such as hypersensitivity reactions and secondary treatment failures, may rarely occur. We report the first case in which repeated BTX‐A injections resulted in both a delayed‐onset cutaneous hypersensitivity reaction and complete secondary treatment failure.

**Case Report/Methods:**

A 42‐year‐old female, with a history of successful BTX‐A treatments for glabellar lines and masseter hypertrophy, experienced diminished efficacy following a treatment session. Ten hours after a touch‐up injection, she developed facial swelling and edematous erythema localized to the injection sites. These manifestations persisted for over one month without any observable aesthetic improvement, indicating complete secondary treatment failure. The therapeutic effect was not restored even after switching to an alternative BTX‐A formulation. We hypothesize that the patient's local hypersensitivity reaction represents a type III immune complex‐mediated response (Arthus reaction) driven by IgG antibodies. The repeated BTX‐A injections may have induced neutralizing IgG antibodies that, in concert with the cutaneous hypersensitivity reaction, contributed synergistically to both the cutaneous reaction and the complete treatment failure. The short interval between the injections may have facilitated these immunologic events.

**Conclusion:**

This case underscores the importance for clinicians to remain vigilant regarding the potential for delayed‐onset cutaneous allergic reactions and complete secondary treatment failure following repeated BTX‐A injections.

## 1 Introduction

Botulinum toxin type A (BTX‐A) injections rank among the most commonly performed cosmetic procedures worldwide. The pharmacological effect of intramuscular BTX‐A is mediated through its binding to receptor sites at motor nerve terminals, resulting in partial chemical denervation of the targeted muscle [1]. The U.S. Food and Drug Administration (FDA) has approved BTX‐A for treating moderate to severe glabellar lines associated with the activity of the corrugator and/or procerus muscles. In addition, off‐label applications of BTX‐A, such as for forehead lines, crow's feet, masseteric hypertrophy, and facial contouring, are widely practiced globally [2].

However, as a heterologous protein derived from bacteria, BTX‐A's immunogenicity remains an ongoing concern. BTX‐A cosmetic injections may induce cutaneous hypersensitivity reactions, although such occurrences are relatively rare [3]. Moreover, a small subset of patients may experience partial secondary treatment failure (PSTF) following multiple sessions—a phenomenon believed to be mediated by the formation of neutralizing antibodies; however, complete secondary treatment failure (CSTF) in cosmetic indications is exceedingly uncommon [4]. Herein, we report a rare case in which a patient developed a prolonged, delayed‐onset cutaneous hypersensitivity reaction that coincided with CSTF following multiple sessions of cosmetic BTX‐A injections.

## 2 Case Presentation

**FIGURE 1:**
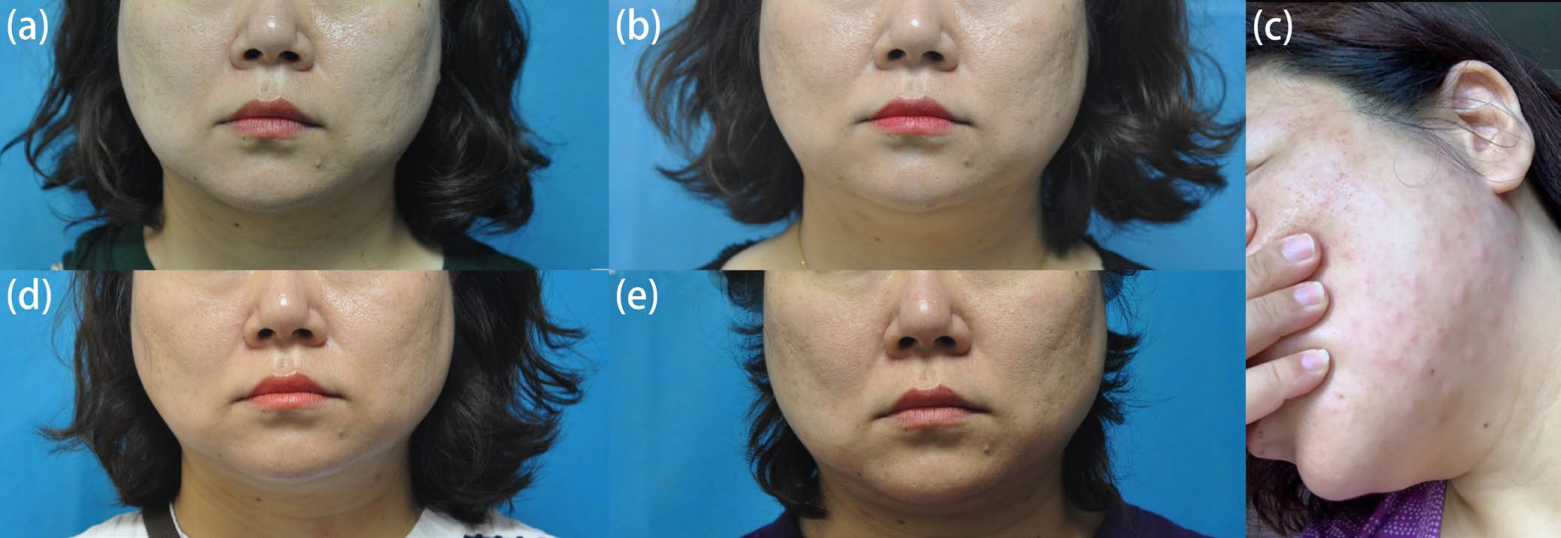
Photographic timeline of treatment progress. (a) Patient on July 21, 2019, before Botox injection. (b) One month post‐injection, showing suboptimal results. (c) Four days after the touch‐up injection, exhibiting facial swelling and erythematous lesions. (d) One month following the touch‐up injection, demonstrating complete secondary treatment failure (CSTF). (e) One month after switching to Hengli injection, indicating persistent CSTF.

A 42‐year‐old female sought cosmetic BTX‐A (Botox, Allergan, Irvine, CA) injection for glabellar lines and masseter hypertrophy at our department on July 21, 2019 (Figure 1a). Over the preceding 5 years, she had received four similar treatments at our department, with dosages ranging from 65 to 70 units per session at intervals spanning 6 months to 3 years, and the patient reported satisfactory results. Her past medical history was unremarkable, and she had no known allergies. In this session, we administered 50 units into the masseter muscles and 20 units into the glabellar region, consistent with her prior treatments. One month later (August 28, 2019), the patient returned, reporting suboptimal results and requesting supplementary treatment (Figure 1b). On examination, her masseter muscles did not exhibit significant atrophy. Accordingly, we provided a touch‐up injection of 30 units into the masseters and 75 units into the platysma band as part of a combined approach to enhance the lower facial contour. However, 10 h post‐treatment, the patient reported facial swelling accompanied by multiple edematous erythematous lesions at the mandibular injection sites (Figure 1c). While a few lesions were pruritic, the majority were asymptomatic. The cutaneous symptoms gradually resolved spontaneously over 3 weeks, yet the facial swelling persisted for over a month; notably, this treatment session produced no aesthetic improvement (Figure 1d).

Five months later (January 22, 2020), the patient returned for further injection. Given her previous cutaneous reaction and CSTF with Botox, we opted to switch to another BTX‐A formulation, Hengli (Lanzhou Institute of Biological Products, China). Additionally, the dosage was increased to 100 units for the bilateral masseter muscles and 80 units for the platysma band. The active ingredient in Hengli is the botulinum neurotoxin type A complex derived from the Hall strain of *Clostridium botulinum* type A—identical to that in Botox—although their excipients differ: Hengli utilizes sucrose, dextran, and gelatin, whereas Botox employs albumin [5, 6]. Despite the absence of any cutaneous hypersensitivity reaction this time, the treatment again failed to yield the desired clinical outcome (Figure 1e). Dissatisfied with the results, the patient declined further examinations or interventions. The timeline of her BTX‐A injections is presented in Table 1.

**TABLE 1:** Timeline of the Patient's BTX‐A Injections.

Session	Date	Sites	Dosage	BTX‐A Formulation	Interval	Treatment result
1	2014.09.05	Masseter and glabellar lines	70u (50u + 20u)	Botox	—	Normal
2	2015.04.12	Masseter and glabellar lines	65u (50u + 15u)	Botox	7 months	Normal
3	2018.04.30	Masseter and glabellar lines	70u (50u + 20u)	Botox	3 years	Normal
4	2019.01.19	Masseter and glabellar lines	70u (50u + 20u)	Botox	9 months	Normal
5	2019.07.21	Masseter and glabellar lines	70u (50u + 20u)	Botox	6 months	PSTF
6	2019.08.28	Masseter and platysmal bands	105u (30u + 75u)	Botox	1 month	CSTF
7	2020.01.22	Masseter and platysmal bands	180u (100u + 80u)	Hengli	5 months	CSTF

Abbreviations: CSTF, complete secondary treatment failure; PSTF, partial secondary treatment failure.

## 3 Discussion

Cutaneous hypersensitivity reactions following cosmetic BTX‐A injections are rare. Studies by Sethi et al., Lee et al. and Nicoletti et al., each involving over 5000 subjects, have reported incidences ranging from 2% to 3%, typically manifesting as transient erythema, edema, or urticaria [7, 8, 9]. In contrast, the present case exhibited erythema and edema that developed 10 h after a touch‐up injection and persisted for more than 1 month before complete resolution, with only a few lesions being pruritic. Notably, this reaction occurred after multiple treatments using the same BTX‐A formulation (Botox), representing an exceedingly rare clinical scenario.

Moreover, the patient experienced CSTF, with no therapeutic effect observed even after switching to an alternative BTX‐A formulation. It is widely accepted that secondary treatment failure (STF) is primarily attributable to the development of IgG antibodies against BTX‐A. These antibodies can be classified as neutralizing (NAbs) and non‐neutralizing antibodies, with the former capable of binding to the receptor sites of the toxin at nerve terminals, thereby inducing treatment resistance or even complete failure [10]. However, the generation of BTX‐A NAb is more frequently encountered in patients with neurological indications and is rare in cosmetic applications; Ho et al. and Rahman et al. have reported NAb incidence rates of 0.2%–0.4%, separately [4, 11]. To date, CSTF following cosmetic injections has been documented only in case reports by Lee et al. and Dressler et al. separately [12, 13]. Given the rarity of both the cutaneous manifestations and the treatment outcome, we postulate that an underlying immunological mechanism may link these phenomena.

To date, no case reports have documented the simultaneous occurrence of cutaneous hypersensitivity and CSTF following BTX‐A injections, as BTX‐A typically remains effective even in the presence of hypersensitivity reactions. Immunologically, this may arise because drug allergies are usually immediate (IgE‐mediated) responses rather than IgG‐mediated reactions.

We therefore hypothesize that the patient's cutaneous hypersensitivity represents a localized type III hypersensitivity reaction (Arthus reaction) to Botox. This reaction typically emerges within hours to days and is mediated by IgG antibody‐driven immune complex formation, which binds to Fc receptors on inflammatory cells and/or activates the complement system, thereby inducing erythema, edema, and pruritus [14]. With repeated Botox injections, the patient may have gradually developed various IgG antibodies against the toxin; some of these antibodies could precipitate a type III cutaneous hypersensitivity reaction, while others—namely, neutralizing antibodies—may lead to STF. These two immunologic events might synergistically enhance immune activation. Moreover, the touch‐up injection administered only 1 month after the previous treatment may have enhanced antigen exposure, stimulating memory B cells to produce high levels of IgG antibodies, thereby amplifying this immune response [14, 15, 16]. We have identified three case reports linking cosmetic BTX‐A injections to type III cutaneous hypersensitivity reactions, all occurring after touch‐up injections. In two of these reports, histopathological analysis confirmed the presence of cutaneous vasculitis [17, 18, 19], supporting the notion that repeated botulinum toxin treatments can induce type III hypersensitivity reactions (see Table 2).

**TABLE 2:** Case Reports of Suspected Cutaneous Type III Hypersensitivity Reactions Following BTX‐A Cosmetic Injections in theLiterature.

	Case 1	Case 2	Case 3
Author, year	Li et al. [17]	Namazi et al. [18]	Namazi et al. [19]
BTX‐A Formulation	Hengli	Canitox	Canitox
Injection sites	Forehead lines and crow's feet	Periorbital and glabellar rhytides	Face (multiple sites)
Injection details	Touch‐up injection (15 days following the preceding injection)	Touch‐up injection (2 weeks following the preceding injection)	Touch‐up injection (2 weeks following the preceding injection)
Onset time	5–11 h	2 h	6 h
Cutaneous manifestations	Impalpable, wine‐purpuric ecchymosis around the injection sites, particularly the upper‐middle face and eyelids, with slight pruritus	Edema, purpuric papules, and erythema in the periorbital region and at the BTX‐A injection sites on the forehead	Edema, erythema, and purpura at the injection sites with dissemination to the lower face and neck
Histopathology	Not available	Vasculitis with panniculitis	Vasculitis
Management	Systemic dexamethasone, vitamin C, and calcium gluconate	Oral prednisolone and hydroxyzine	Oral prednisolone
Duration	96 h; residual hyperpigmentation at 1 month‐ follow‐up	2 weeks	2 weeks

To the best of our knowledge, this is the first case report documenting the simultaneous occurrence of a delayed‐onset cutaneous hypersensitivity reaction and CSTF following multiple BTX‐A injections. Ideally, we would have conducted a skin biopsy, Botox intradermal test, skin prick and patch tests, as well as mouse protection/diaphragm assays, ELISA, and Western blot analyses to elucidate the underlying etiology further [12, 13, 20, 21, 22]. Unfortunately, due to the patient's dissatisfaction with prior treatment outcomes, she declined further diagnostic evaluations. Current perspectives also indicate that the immunogenicity of botulinum toxin complexes primarily arises from their complexing proteins rather than the neurotoxin itself. Notably, after switching to Hengli, the patient did not present any cutaneous hypersensitivity reaction despite the continuing STF. This raises the possibility that she may have been allergic to the excipients (albumin) or bacterial impurities in Botox [23]. In her case, transitioning to incobotulinumtoxinA—which contains only the 150 kDa neurotoxin without any complexing proteins—might have restored therapeutic efficacy, though this benefit could be limited [22]. However, we could not pursue such a clinical strategy because only Botox and Hengli are approved in China.

In any event, clinicians should be vigilant regarding the potential for delayed‐onset cutaneous hypersensitivity reactions following repeated BTX‐A injections and remain alert to the possibility that such allergic responses may promote the production of neutralizing antibodies, ultimately leading to CSTF. Given the widespread use of BTX‐A injections in aesthetic medicine, further research into the immunogenicity and allergenic potential of BTX‐A is warranted to enhance its clinical safety and effectiveness.

## Author Contributions

All authors have reviewed and approved the article for submission. Conceptualization: Yan Bian and Hong Cai. Writing – original draft preparation: Chi Zhang, Shaohua Wang, and Lili Zhang. Writing – review and editing: Chi Zhang. Visualization: Yan Bian. Supervision: Hong Cai.

## Ethics Statement

This study was conducted under the principles outlined in the Declaration of Helsinki. All procedures performed were in compliance with ethical standards and guidelines for medical research involving human subjects. Written informed consent was obtained from the patient for participation in the study and for publication of the case details and accompanying images, with explicit permission granted for image reuse.

## Conflicts of Interest

The authors declare no conflicts of interest.

## Data Availability

The data that support the findings of this study are available from the corresponding author upon reasonable request.
